# Community diversity, structure and carbon footprint of nematode food web following reforestation on degraded Karst soil

**DOI:** 10.1038/srep28138

**Published:** 2016-06-17

**Authors:** Ning Hu, Hui Li, Zheng Tang, Zhongfang Li, Jing Tian, Yilai Lou, Jianwei Li, Guichun Li, Xiaomin Hu

**Affiliations:** 1 College of Resources and Civil Engineering, Northeastern University, 110819 Shenyang, China; 2 Institute of Environment and Sustainable Development in Agriculture, Chinese Academy of Agricultural Sciences, 100081 Beijing, China; 3 College of Resources and Environmental Sciences, Henan Agricultural University, 450002 Zhengzhou, China; 4 College of Chemistry and Bioengineering, Hezhou University, 542899 Hezhou, China; 5 Key Laboratory of Ecosystem Network Observation and Modeling, Institute of Geographic Sciences and Natural Resources Research, Chinese Academy of Sciences, 100101 Beijing, China; 6 National Engineering Laboratory for Improving Quality of Arable Land, Institute of Agricultural Resources and Regional Planning, Chinese Academy of Agricultural Sciences, 100081 Beijing, China

## Abstract

We examined community diversity, structure and carbon footprint of nematode food web along a chronosequence of *T. Sinensis* reforestation on degraded Karst. In general, after the reforestation: a serious of diversity parameters and community indices (Shannon-Weinier index (H′), structure index (SI), etc.) were elevated; biomass ratio of fungivores to bacterivores (FFC/BFC), and fungi to bacteria (F/B) were increased, and nematode channel ratio (NCR) were decreased; carbon footprints of all nematode trophic groups, and biomass of bacteria and fungi were increased. Our results indicate that the Karst aboveground vegetation restoration was accompanied with belowground nematode food web development: increasing community complexity, function and fungal dominance in decomposition pathway, and the driving forces included the bottom-up effect (resource control), connectedness of functional groups, as well as soil environments.

Karst terrains formed from carbonate minerals account for about 15% of the world’s land area^1^. The unique ecosystem is very fragile and sensitive, with a low environmental capacity^2^, and the soil is thin, coarse, highly erosive and degenerative^3^. Karst soil covering approximately 0.55 million km^2^ in southwest China, has been subjected to intensive anthropogenic disturbances (e.g., cultivation, deforestation, grazing and burning)^4^,^5^. These disturbances rapidly expand after 1970s because of the pressure from the increasing population and land overuse^6^,^7^. This accelerates Karst ecosystem degradation especially of rocky-desertification^8^. The degradation adversely affects soil fertility and results in the genesis of abandoned bare land. Such degraded land needs proper ecological restoration through which soil can be ameliorated to support biological productivity^9^. Reforestation, as one method by which degraded sites can be restored back to maintain soil fertility^9^, has been increasingly adopted in Chinese Karst regions since 1990s, and *T. Sinensis* is one of the commonly used tree species.

Soil nematode communities are useful biological indicators of soil health, because they form a dominant group of soil organisms and live in various types of soils^10^. These communities also represent key links in soil food webs, such as plant-parasite, bacterivores, fungivores, omnivores and predators, and their trophic structures are closely correlated with soil ecosystem processes^10^. Nematode community size and complexity can reflect the vegetation change and are regarded as sensitive bio-indicators of ecosystem restoration^11^. Microbiovorous nematodes, as consumers of microflora, indirectly regulate decomposition and release of nutrients in ecosystems^10^. The relative importance of bacterivores vs. fungivores is closely related to that of bacteria vs. fungi, and the relevant indices such as fungivores to bacterivores ratio (FF/BF) and nematode channel ratio (NCR) are extensively used to indicate the decomposition pathway of soil food web^12^. Recently, there has been an increasing interest in the metabolic activity of nematode communities, which provides information on the magnitude or nature of ecosystem functions^13^,^14^,^15^. Ferris suggested using the nematode metabolic footprint to extend ecosystem assessments^13^. This footprint conveys additional information on the biomass, metabolic activity, and magnitudes of carbon (C) and energy flow in soil food webs, and provides an effective method for monitoring the available resources and estimating the contribution of nematodes to ecosystem services and functions^11^.

Recently, nematode community characteristics were reported in the Karst regions^16^,^17^,^18^. However, to date, nematodes have been poorly known following *T. Sinensis* reforestation on the degraded Karst soil. Reforestation can increase vegetation diversity and belowground resource input and can improve soil environments such as moisture and porosity^2^,^19^. Consequently, the *T. Sinensis* reforestation on Karst may increase nematode community size, complexity and carbon footprint. Additionally, the *T. Sinensis* reforestation can alter the type of resource input, and therefore may change the resource quality and in turn may influence nematode food web structure and decomposition pathway. The main objective of this study was to examine diversity, structure and carbon footprint of nematode food web in response to *T. Sinensis* reforestation on the degraded Karst soils.

## Results

### Soil basic properties

There were significant differences in all the studied soil environmental variables (except for pH) among the different ages of forests (*P* < 0.05) (Table 1). These variables showed an increasing trend with the reforestation age. To the 16-yr age, soil porosity, moisture, TOC content, TN content, and C:N increased by 28.1%, 42.0%, 46.9%, 13.2% and 30.3%, respectively, compared with the 0-yr control.

### Nematode community composition

**Figure 1:**
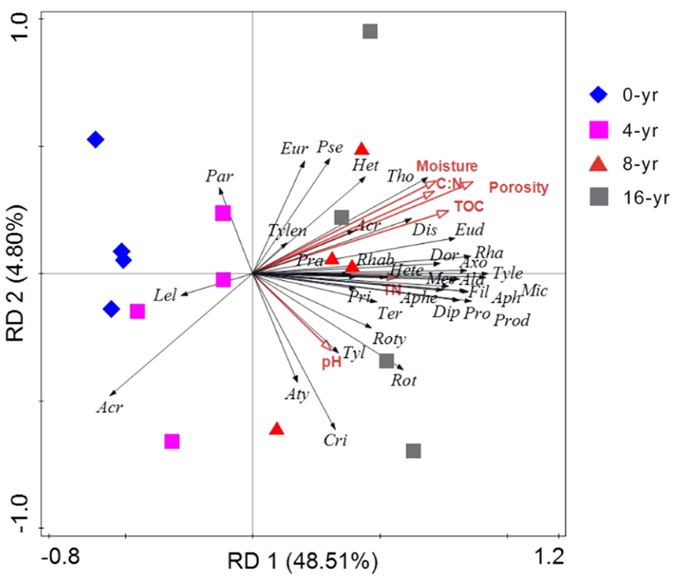
Redundancy analysis (RDA) of nematode genera in relation to soil environmental variables. Nematode genus abbreviations were shown in Table 1.

At the 0-yr control site, some genera (such as *Alaimus*, *Tylencholaimus*, *Rotylenchulus*, *Axonchium*, *Microdorylaimus and Mesodorylaimus*) were inexistent and only 23 genera were recorded (Table 2). After reforestation, these inexistent genera gradually appeared and the recorded genera number increased to 34 of the 16-yr reforestation. The dominant genera were *Acrobeloides, Rhabdolaimus* and *Aphelenchoides* for all the four study sites, and their relative abundances all decreased with the reforestation age. A redundancy analysis (RDA) showed that the composition of nematode community was clearly discriminated among the different ages of forests, and showed that soil basic properties of moisture, porosity and TOC were closely related to the distribution of nematode genera (Fig. 1). The eigenvalues were 0.131 (*F* = 3.412, *P* = 0.003) and 0.242 (*F* = 2.157, *P* = 0.002) for the first canonical axis and all canonical axes, respectively, and the first two axes explained 53.3% of the variation.

### Nematode diversity and food web structure

All measured community indices significantly differed among the four study sites in both seasons (*P* < 0.05) (Table 3). The values of H′, SR, and J′ all tended to increase with the reforestation age, and to the 16-yr reforestation increased by 44.9%, 41.0% and 19.4%, respectively, in June, and by 32.3%, 38.2% and 16.0%, respectively, in September. The values of MI and SI were both greater in the older forests than the younger forests in both seasons. After the 16-yr reforestation the NCR value decreased by 28.2%, compared with the 0-yr control in both seasons. The FF/BF ratio and F/B ratio both showed an increasing tendency with the reforestation age in both seasons.

### Community size and biomass carbon

**Figure 2:**
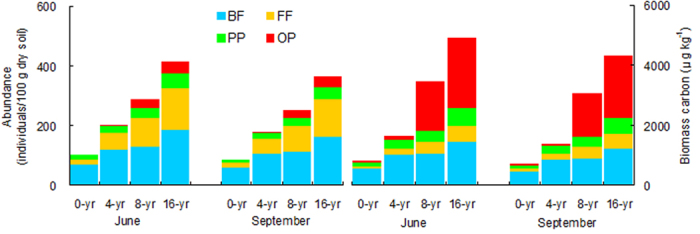
Mean abundances and biomass carbon of different nematode groups along a choronosequence of *T. Sinensis* reforestation (*n* = 4). BF, FF, PP, OP: bacterivores, fungivores, plant-parasite, omnivores-predators, respectively.

**Figure 3:**
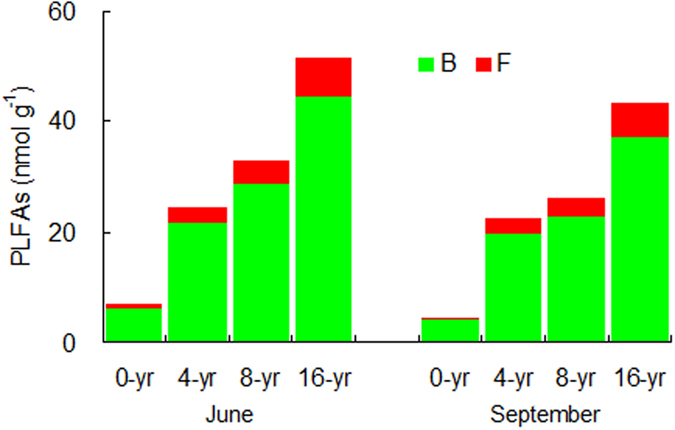
Mean phospholipid fatty acids (PLFAs) contents of bacteria (B) and fungi (F) along a choronosequence of *T. Sinensis* reforestation (*n* = 4).

**Figure 4:**
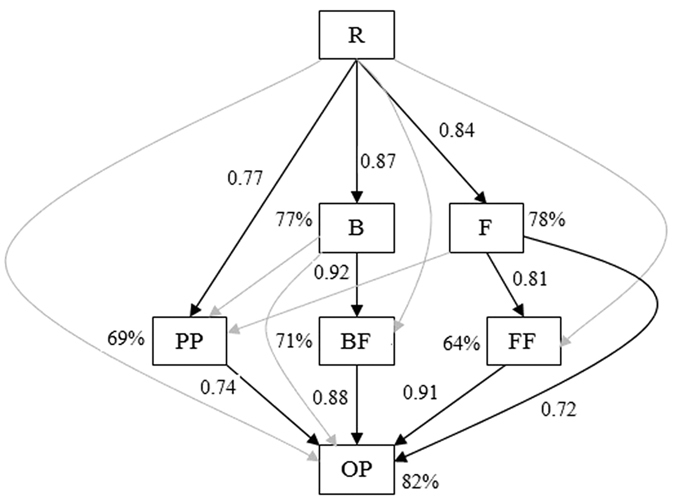
Structural equation model of bottom-up effect of the nematode food web (*x*^2^ = 8.472, df = 14, *P* = 0.649, CFI = 0.988, IFI = 0.971, RMSEA = 0.002). Numbers next to the arrows are the standardized path coefficients. Black arrows indicate significant relationships (*P* < 0.05). Grey arrows indicate non-significant relationships or paths removed to optimize model fits. Percentages close to endogenous variables indicate the variance explained by the model. R: root biomass; B and F: biomass of bacteria and fungi, respectively; BF, FF, PP and OP: biomass carbon of bacterivores, fungivores, plant-parasites and omnivores-predators, respectively.

In general, the abundance and biomass carbon of all nematode trophic groups significantly increased with the reforestation age (*P* < 0.05) (Fig. 2). On average, the abundance and biomass carbon of total nematode increased at a rate of 19 individuals 100 g^−1^ yr^−1^ and 267 μg kg^−1^ yr^−1^, respectively, in June, and of 17 individuals 100 g^−1^ yr^−1^ and 236 μg kg^−1^ yr^−1^, respectively, in September. The consistent increasing trend was also found for the biomass of bacteria, fungi, and total microbes in both seasons (Fig. 3). The final structural equation model (SEM) on the bottom-up effect of the nematode food web adequately fit the data and the standardized path coefficients (*x*^2^ = 8.472, df = 14, *P* = 0.649, CFI = 0.988, IFI = 0.971, RMSEA = 0.002) (Fig. 4). Root biomass (R) was significantly correlated to the biomass of bacteria (B), fungi (F), and plant-parasites (PP); the B and F were significantly correlated to the biomass of bacterivores (BF) and fungivores (FF), respectively; and the BF, FF, F and PP were all significantly correlated to the biomass of omnivores-predators (OP). The model explained 71%, 64%, 69% and 82% of the variance in BF, FF, PP, and OP, respectively.

## Discussion

In our study, a RDA analysis indicated that the nematode communities of different sites clustered corresponding to forest age. The nematode community composition change might be directly related to the vegetation restoration. The vegetation coverage and diversity increased after the *T. Sinensis* reforestation in our study. Additionally, soil basic properties can control nematode communities^20^,^21^. Our RDA analysis showed that soil TOC content, porosity and moisture were closely related to nematode genera distribution. In the present study, a serious nematode ecological indices (genus number, species richness index (SR), evenness index (J′) and Shannon-Weiner diversity index (H′), maturity index (MI), and structure index (SI)) generally increased with the reforestation age. These results indicated that the *T. Sinensis* reforestation created the more diverse and structured nematode community. The *T. Sinensis* reforestation increased belowground resource input (as indicated by the elevated root biomass) and improved soil environments (as suggested by the increased porosity and moisture, etc.), and therefore the relatively complicated and mature soil food web was developed^22^. Additionally, generally there is a good association of aboveground and belowground biodiversity^23^,^24^. The increased nematode diversity might be directly resulted from the increased vegetation spices diversity in out study. Consistently with our results, Guan *et al*.^25^ showed a gradually increasing trend in nematode community complexity with increasing age of *Caragana microphylla*. However, another study reported a different pattern that the most complicated nematode community was not found in the old but the mid-age forest. Kardol *et al*.^26^ showed that restoration of aboveground communities is of limited indicative value for belowground developments: successful restoration of vegetation diversity does not necessarily imply successful restoration of belowground diversity. The different patterns of nematode diversity in a vegetation choronosequence may depend on different vegetation types, time scales, and study sites.

The extent to which decomposition is fungal-mediated can be reflected by the structure of the microbial feeding nematode community^27^,^28^. In our study, as expected, the nematode channel ratio (NCR) tended to decline with forest age, indicating a shift relatively towards fungal-dominant decomposition pathway. The increased ratio of carbon footprint of fungivores to bacterivores (FF/BF) also suggested a greater flow of resources into the food web through fungivorous channels than bacterivorous channels in older forests. The biomass ratio of fungi to bacteria (F/B) also showed the increasing trend. This can be explained by the change in the belowground resource quality. In our study, the lignin content and C/N ratio of the root tended to increase, while N content tended to decrease after the reforestation. This indicated that the more recalcitrant resource was formed and this therefore favored the fungal dominance in decomposition channel. Increasing soil aggregation is usually associated with greater fungal activity^29^,^30^. It is known that fungi are aerobic organisms^30^, and thus great aeration may favor fungal-mediated decomposition^31^. In our investigation, the increased soil porosity means the increased soil aggregation and aeration, and this so might contribute to the growing importance of fungal mediation in decomposition to some extent after the reforestation. Similar decomposition pathway results were also reported in other studies^12^,^26^,^32^,^33^,^34^. These findings were in agreement with succession from bacterivory to fungivory often found in nematode faunas^35^,^36^ and with a general view of predominance of a fungus-based decomposition channel in advanced successional stages^37^.

Consistently with abundance, biomass carbon of total nematode and trophic groups all increased with reforestation age in our study. The nematode trophic biomass indicated the C and energy flow into the soil food web through their respective trophic channels^13^. The biomass of bacteria, and fungi also increased. These results demonstrated that after the *T. Sinensis* reforestation the community size and function of the nematode food web. The bottom-up effect (resource control) generally occurs in soil food web^11^,^38^. The predator-prey channel was one of the primary channels of the soil micro-food web^39^, and the flow of C and energy through the soil food web is mainly driven by the feeding interrelationship among soil biota communities^38^,^40^. In our structural equation model (SEM), root biomass (R) directly affected the biomass of bacteria (B), fungi (F), and plant-parasites (PP); the B and F significantly affected the biomass of bacterivores (BF) and fungivores (FF), respectively; and the F, BF, FF and PP together influenced the biomass of omnivores-predators (OP). These data confirmed that bacterivores and fungivores both played important roles in the C and energy flow between microbes and nematodes, and bacteria and fungi acted as primary prey for bacterivores and fungivores, respectively; that fungi, bacterivores, fungivores and plant-parasites were primary prey for omnivores-predators; and that the resource input controlled the nematode food web. The variance in the food web unexplained by the model possibly attributed to soil environmental variables such as moisture and porosity. Our results indicate that the bottom-up effect from the vegetation, the interactions of the functional groups, as well as soil environments together were the drivers of the reforested Karst soil food web.

## Methods

### Study site and experiment design

This study was conducted at Huanjiang county (107°54′ E, 24°49′ N), Guangxi province in China, with typical Karst ecosystems. This region belongs to a subtropical monsoon climate with mean annual precipitation of 1,389 mm and mean annual temperature of 19.9 °C. The calcareous soil developed from a dolostone base^41^. The soil degradation is serious due to the intensive anthropogenic disturbance especially of cultivation and therefore results in a large area of abandoned bare land in this region. Since 1990s, large scales of *T. Sinensis* forestation have been adopted to restore the degraded ecosystem. Three *T. Sinensis* forestations (4-, 8-, and 16-yr) were selected as the experimental sites while a nearby abandoned bare land as the 0-yr control site. The four sites were selected to show very similar altitude, topography, vegetation background and disturbance experience (over 40 years of cultivation) before forestation and the same soil type. Each site has a size of over 10,000 m^2^, and four plots (40 m × 40 m) as replications were selected within each site. The distance between plots within a site was about 10 m. The *T. Sinensis* was planted at a 7 m × 5 m density. We investigated characteristics of vegetation and belowground resources in June 2015. The tree diameter and height averaged 5 cm and 2.54 m, 11 cm and 2.59 m, 19 cm and 2.68 m for the 4-, 8-, and 16-year forestations, respectively. The vegetation species diversity and coverage degree showed an increasing trend with the reforestation age (Table 4). The resource input for the belowground soil food web was mainly from the turnover of the roots (including the fine roots of the shrub and tree and the herbaceous root). We used the excavation method (10 cm × 10 cm × 10 cm) to collect the roots, and then measured the root biomass and chemistry. The root cellulose and lignin contents were measured using the colorimetric method and Klason method, respectively^42^, and the root C and N contents were determined using an element Analyzer (Elementar, Germany). The root biomass significantly increased with the reforestation age, and the root chemistry notably differed among the sites: the lignin content and C/N tended to increase, while the N content tended to decrease with the reforestation age (Table 4). Ten soil cores (2.5 cm diameter) were collected to a depth of 10 cm depth along an S-shaped transect using an auger and were mixed to form one composite sample, after excluding litter and humus layer from each plot in both seasons of June and September 2015.

### Nematode community analysis

Nematodes were extracted from 100 g field moist soil by a modified cotton-wool filter method^11^. After counting the total number of nematodes in a sample, 100 individuals were randomly selected and identified to genus level using an inverted compound microscope according to Bongers^43^ and Ahmad and Jairjpuri^44^. If the total nematodes did not reach 100 in a sample, all the nematodes in the sample were identified. Nematode abundance was expressed as individuals per 100 g of dry soil. Nematodes were assigned to the following trophic groups according to their feeding habits: bacterivores (BF), fungivores (FF), omnivores-predators (OP) and plant-parasites (PP)^45^.

Following identification, the length (*L*) and maximum body diameter of all nematodes were measured using an ocular micrometer. Nematode biomass was calculated using the following formula:


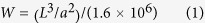


where *W* is the fresh weight (μg) per taxon, *L* is nematode length (μm), and *a* is the ratio of length to maximum body diameter^45^. Total nematode biomass C was estimated by multiplying the abundance of each taxon by their calculated fresh weight^46^, using a fresh weight/dry weight conversion factor of 0.20^47^ and a C content of 52% of dry weight^48^.

The Shannon-Weiner diversity index (H′), species richness index (SR) and evenness index (J′) were used as the indication of soil nematode diversity and were calculated using the following formulae:


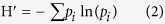


where *p*_*i*_ is the proportion of individuals in the *i*th taxon^49^;


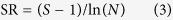


where *S* is the total number of genera and *N* is the total number of individuals in the community^50^;


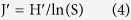


where H′ is Shannon-Weinier index and *S* is the total number of genera^51^. Community indices of nematode channel ratio (NCR), maturity index (MI) and structure index (SI) were calculated as follows:


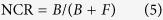


where *B* and *F* are the numbers of bacterivores and fungivores in the total nematode community, respectively^12^;


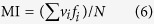


where *v*_*i*_ is the c-p value assigned to genus *i*, *f*_*i*_ is the frequency of genus *i*, and *N* is the total number of individuals in the community^52^;


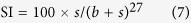


where *b* and *s* are the abundance of individuals in guilds in the basal component and structural component weighted by their *k*_*b*_ and *k*_*s*_ values, respectively. *k*_*b*_ is the weighting assigned to guilds Ba_2_ and Fu_2_, and *k*_*s*_ is the weighting assigned to guilds Ba_3_–Ba_5_, Fu_3_–Fu_5_, and Op_3_–Op_5_. Ba_*x*_, Fu_*x*_, Op_*x*_, and Pp_*x*_ (where *x* = 1–5) represent the functional guilds of nematodes that are bacterivores (Ba), fungivores (Fu), omnivores-predators (Op) or plant parasites (Pp), respectively, where the guilds have the character indicated by *x* on the colonizer-persister (c-p) scale (1–5) according to their *r* and *K* characteristics.

### Measurement of microbes and soil basic properties

Phospholipid fatty acids (PLFAs) analysis was performed using the method described by Helgason *et al*.^53^. Briefly, fatty acids were extracted from 4 g freeze-dried soils using a single phase chloroform, methanol, phosphate buffer solution. The isolated fatty acid methyl esters (FAMEs) were analyzed by a gas-chromatography mass-spectroscopy system (TRACE GC Ultra ISQ, Thermo Fisher Scientific) using a DB-5 column with 30 m length, 0.25 mm I.D., and 0.25 μm film thickness. Helium was used as a carrier gas. The temperature program started at 150 °C for 4 min, thereafter the temperature was ramped to 250 °C at a rate of 4 °C min^−1^ and held 5 min. The PLFAs were identified by a comparison of retention times to known standards (FAME 37 47885-U, Supelco, Inc.) and a standard bacterial acid methyl ester mixture (BAME 26 47080-U, Supelco, Inc.)^54^. The contents of PLFAs (nmol g^−1^ dry soil) were quantified based on the internal standard methyl nonadecanoate (19:0). The sum of thirteen PLFAs (i15:0, a15:0, 15:0, i16:0, 16:1ω9, 16:1ω7t, i17:0, a17:0, 17:0, cy17:0, cy19:0, 18:2ω6 and 18:1ω9c) served as a measure of total microbial biomass (nmol g^−1^). PLFAs i15:0, a15:0, 15:0, i16:0, 16:1ω9, 16:1ω7t, i17:0, a17:0, 17:0, cy17:0 and cy19:0 referred to the bacterial biomass^55^,^56^. Fatty acids 18:2ω6 and the isomer 18:1ω9c were used as indicators for the fungal biomass^57^. We also calculated the ratio of fungal to bacterial PLFAs (F/B).

Subsamples collected in June were used to measure soil basic properties: porosity was determined using a core method based on undisturbed soil^58^; moisture was measured by an oven-dry method, and expressed on mass basis of dry soil; total organic carbon (TOC) was measured using a K_2_Cr_2_O_7_ oxidation method^58^; total nitrogen (TN) was measured using an element Analyzer (Elementar, Germany), and the TOC to TN ratio (C:N) was calculated; soil pH was measured with a pH meter.

### Data analysis

Nematode abundances were ln (*x* + 1) transformed prior to statistical analysis to obtain normality of data. Using SPSS 13.0 software, all data were subjected to a one-way analysis of variance (ANOVA) with a LSD test to evaluate the reforestation age effect in both seasons. Differences at *P* < 0.05 were considered statistically significant. The relationship between nematode genera (seasonally mean data) and soil environmental variables was examined based on a redundancy analysis (RDA) using the CANOCO software. A structural equation modeling (SEM) was used to evaluate the bottom-up effect (resource control) and the connectedness of the functional groups of the nematode food web, according to documents on the interactions of the following variables^11^,^14^,^39^,^59^: root biomass (R), the biomass of bacteria (B) and fungi (F), and the biomass carbon of bacterivores (BF), fungivores (FF), plant parasites (PP) and omnivores-predators (OP). The analysis was performed with AMOS 7.0 software using the ‘robust’ maximum likelihood estimation procedures.

## Additional Information

**How to cite this article**: Hu, N. *et al*. Community diversity, structure and carbon footprint of nematode food web following reforestation on degraded Karst soil. *Sci. Rep.* **6**, 28138; doi: 10.1038/srep28138 (2016).

## Figures and Tables

**Table 1:** Soil basic properties along a choronosequence of *T. Sinensis* reforestation (mean ± standard deviation, *n* = 4).

	0-yr	4-yr	8-yr	16-yr
Porosity (%)	31.41 ± 2.65 c	33.62 ± 2.61 bc	38.41 ± 3.42 ab	40.24 ± 3.10 a
Moisture (%)	11.42 ± 1.74 c	11.91 ± 2.23 bc	15.12 ± 1.86 ab	16.22 ± 1.83 a
TOC (g kg^−1^)	11.94 ± 1.17 c	13.12 ± 1.20 bc	15.61 ± 3.32 ab	17.54 ± 2.21 a
TN (g kg^−1^)	1.21 ± 0.10 b	1.22 ± 0.07 b	1.29 ± 0.13 ab	1.37 ± 0.05 a
C:N	9.85 ± 0.53 b	10.74 ± 1.21 ab	12.12 ± 1.83 ab	12.83 ± 1.69 a
pH	6.72 ± 0.28	6.79 ± 0.17	6.91 ± 0.21	6.82 ± 0.09

Different letters indicate significant difference between treatments at *P* < 0.05. TOC, total organic carbon; TN, total nitrogen; C:N, ratio of TOC to TN.

**Table 2:** Seasonally mean relative abundance (%) of nematode genera along a choronosequence of *T. Sinensis* reforestation.

Genus	Abbr.	0-yr	4-yr	8-yr	16-yr
Bacterivores					
*Diplogasteriana*	*Dip*	0.7	1.2	1.9	2.9
*Protorhabditis*	*Pro*	0.9	2.4	2.4	3.3
*Rhabditonema*	*Rha*	0.4	1.9	2.2	3.6
*Acrobeles*	*Acr*	6.4	2.2	1.1	0.9
*Heterocephalobus*	*Het*	1.5	1.2	0.9	1.8
*Pseudacrobeles*	*Pse*	1.5	1.3	1.2	1.6
*Teratocephalus*	*Ter*	1.5	1.4	0.7	1.6
*Eucephalobus*	*Eur*	0.8	0.7	0.7	1.1
*Acrobeloides*	*Acr*	21.2	18.7	12.6	10.1
*Tylocephalus*	*Tyl*	1.2	1.0	0.9	1.1
*Prismatolaimus*	*Pri*	0.9	0.7	1.5	0.9
*Rhabdolaimus*	*Rhab*	29.1	23.0	14.3	11.2
*Alaimus*	*Ala*	0.0	2.0	2.2	2.3
Fungivores					
*Aphelenchus*	*Aph*	1.9	5.9	7.9	9.6
*Aphelenchoides*	*Aphe*	16.7	15.9	15.2	10.4
*Filenchus*	*Fil*	1.4	5.5	7.8	8.4
*Tylencholaimus*	*Tyle*	0.0	0.6	4.2	6.7
Plant-parasites					
*Atylenchus*	*Aty*	2.3	1.8	1.4	0.7
*Lelenchus*	*Lel*	2.3	1.8	1.1	0.6
*Tylenchus*	*Tylen*	1.9	1.7	1.1	0.5
*Paratylenchus*	*Par*	2.2	1.7	1.2	0.6
*Criconemella*	*Cri*	0.0	0.5	0.4	0.5
*Pratylenchus*	*Pra*	0.8	0.8	0.5	1.1
*Rotylenchulus*	*Rot*	0.0	0.0	0.5	1.0
*Rotylenchus*	*Roty*	0.0	0.0	0.6	0.7
*Heterodera*	*Hete*	2.6	2.8	2.2	2.1
*Axonchium*	*Axo*	0.0	0.9	1.3	1.8
*Dorylaimellus*	*Dor*	0.0	1.1	1.3	1.9
Omnivores-Predators					
*Thonus*	*Tho*	0.8	0.7	1.5	1.6
*Eudorylaimus*	*Eud*	0.9	0.7	1.6	2.1
*Microdorylaimus*	*Mic*	0.0	0.0	1.8	1.8
*Discolaimus*	*Dis*	0.0	0.0	0.0	1.6
*Mesodorylaimus*	*Mes*	0.0	0.0	1.6	1.9
*Prodorylaimium*	*Prod*	0.0	0.0	4.2	2.0

**Table 3:** Indices of nematode diversity and food web structure along a choronosequence of *T. Sinensis* reforestation (mean ± standard deviation, *n* = 4).

		0-yr	4-yr	8-yr	16-yr
June	H′	2.02 ± 0.13 d	2.35 ± 0.15 c	2.75 ± 0.07 b	2.93 ± 0.10 a
	SR	3.22 ± 0.43 b	3.62 ± 0.47 b	4.51 ± 0.13 a	4.54 ± 0.40 a
	J′	0.74 ± 0.02 b	0.78 ± 0.03 b	0.84 ± 0.02 a	0.88 ± 0.03 a
	MI	2.25 ± 0.07 b	2.30 ± 0.03 b	2.44 ± 0.09 a	2.42 ± 0.08 a
	SI	56.8 ± 8.81 b	56.1 ± 1.71 b	69.6 ± 5.14 a	71.5 ± 6.42 a
	NCR	0.78 ± 0.05 a	0.69 ± 0.07 a	0.57 ± 0.03 b	0.56 ± 0.06 b
	FF/BF	0.14 ± 0.03 c	0.22 ± 0.04 b	0.37 ± 0.04 a	0.39 ± 0.06 a
	F/B	0.10 ± 0.01 c	0.14 ± 0.02 b	0.14 ± 0.01 b	0.16 ± 0.01 a
September	H′	2.26 ± 0.14 c	2.54 ± 0.13 b	2.81 ± 0.17 ab	2.99 ± 0.16 a
	SR	4.14 ± 0.41 c	5.02 ± 0.34 b	5.45 ± 0.42 ab	5.72 ± 0.36 a
	J′	0.81 ± 0.04 b	0.84 ± 0.03 b	0.91 ± 0.03 a	0.94 ± 0.03 a
	MI	2.44 ± 0.09 b	2.66 ± 0.11 a	2.74 ± 0.14 a	2.78 ± 0.12 a
	SI	62.1 ± 7.94 b	64.2 ± 4.56 b	73.6 ± 4.26 a	74.6 ± 5.51 a
	NCR	0.71 ± 0.05 a	0.61 ± 0.04 b	0.53 ± 0.04 bc	0.51 ± 0.05 c
	FF/BF	0.15 ± 0.02 c	0.24 ± 0.03 b	0.39 ± 0.04 a	0.40 ± 0.05 a
	F/B	0.10 ± 0.01 c	0.14 ± 0.01 b	0.17 ± 0.01 a	0.17 ± 0.02 a

Different letters indicate significant difference between treatments at *P* < 0.05. H′, Shannon diversity index; SR, spices richness index; J′, evenness index; MI, maturity index; SI, structure index; NCR, nematode channel ratio; FF/BF, ratio of biomass carbon of fungivores to bacterivores; F/B, ratio of fungal to bacterial biomass.

**Table 4:** Information on aboveground vegetation and belowground resource input (0–10 cm depth) along a choronosequence of *T. Sinensis* reforestation (mean ± standard deviation, *n* = 4).

	0-yr	4-yr	8-yr	16-yr
Vegetation				
Spices number (100 m^−2^)	3.62 ± 0.25 d	12.41 ± 1.02 c	18.55 ± 1.46 b	22.25 ± 1.79 a
Cover degree (%)	5.12 ± 0.35 c	34.66 ± 2.12 b	37.51 ± 3.12 b	46.31 ± 3.75 a
Belowground resource				
Root biomass (g m^−2^)	41.47 ± 5.17 c	291.1 ± 28.2 b	328.2 ± 29.4 b	416.1 ± 27.2 a
C content (%)	40.21 ± 3.12	41.22 ± 2.97	44.31 ± 4.12	41.15 ± 3.01
N content (%)	1.42 ± 0.12 a	1.04 ± 0.09 b	0.86 ± 0.04 c	0.82 ± 0.05 c
C/N	28.32 ± 2.51 c	39.63 ± 2.77 b	51.52 ± 4.81 a	50.18 ± 4.64 a
Cellulose content (%)	26.72 ± 1.58	26.19 ± 2.23	24.92 ± 2.19	27.82 ± 2.46
Lignin content (%)	12.41 ± 1.21 c	21.56 ± 1.74 b	22.22 ± 1.88 b	29.47 ± 2.14 a

Different letters indicate significant difference between treatments at *P* < 0.05.
